# Walker 256 Tumor Growth Suppression by Crotoxin Involves Formyl Peptide Receptors and Lipoxin A_4_

**DOI:** 10.1155/2016/2457532

**Published:** 2016-04-12

**Authors:** Patrícia Brigatte, Odair Jorge Faiad, Roberta Cornélio Ferreira Nocelli, Richardt G. Landgraf, Mario Sergio Palma, Yara Cury, Rui Curi, Sandra Coccuzzo Sampaio

**Affiliations:** ^1^Special Laboratory of Pain and Signaling, Butantan Institute, Avenida Vital Brazil 1500, 05503-900 São Paulo, SP, Brazil; ^2^CEIS/Department of Biology, Institute of Biosciences of Rio Claro, São Paulo State University (UNESP), Rio Claro, SP, Brazil; ^3^Laboratory of Pathophysiology, Butantan Institute, Avenida Vital Brazil 1500, 05503-900 São Paulo, SP, Brazil; ^4^Department of Natural Sciences, Mathematics and Education, Agricultural Sciences Center, Federal University of São Carlos, Rodovia Anhanguera Km 174, 13600-970 Araras, SP, Brazil; ^5^Laboratory of Inflammation and Vascular Pharmacology, Federal University of São Paulo, Rua São Nicolau 210, 09913-030 Diadema, SP, Brazil; ^6^Department of Physiology and Biophysics, Institute of Biomedical Sciences, University of São Paulo, Avenida Professor Lineu Prestes 1524, 05508-900 São Paulo, SP, Brazil; ^7^Department of Pharmacology, Institute of Biomedical Sciences, University of São Paulo, Avenida Professor Lineu Prestes 1524, 05508-900 São Paulo, SP, Brazil

## Abstract

We investigated the effects of Crotoxin (CTX), the main toxin of South American rattlesnake (*Crotalus durissus terrificus*) venom, on Walker 256 tumor growth, the pain symptoms associated (hyperalgesia and allodynia), and participation of endogenous lipoxin A_4_. Treatment with CTX (s.c.), daily, for 5 days reduced tumor growth at the 5th day after injection of Walker 256 carcinoma cells into the plantar surface of adult rat hind paw. This observation was associated with inhibition of new blood vessel formation and decrease in blood vessel diameter. The treatment with CTX raised plasma concentrations of lipoxin A_4_ and its natural analogue 15-epi-LXA_4_, an effect mediated by formyl peptide receptors (FPRs). In fact, the treatment with Boc-2, an inhibitor of FPRs, abolished the increase in plasma levels of these mediators triggered by CTX. The blockage of these receptors also abolished the inhibitory action of CTX on tumor growth and blood vessel formation and the decrease in blood vessel diameter. Together, the results herein presented demonstrate that CTX increases plasma concentrations of lipoxin A_4_ and 15-epi-LXA_4_, which might inhibit both tumor growth and formation of new vessels via FPRs.

## 1. Introduction

Crotoxin (CTX) is the main toxic component of the venom of the South American rattlesnake,* Crotalus durissus terrificus* [1, 2]. The toxin is a heterodimeric complex consisting of basic and toxic phospholipase A_2_ and an acidic, nontoxic, and nonenzymatic component named crotapotin. In addition to its toxic properties, several experimental observations indicated that CTX also has immunomodulatory, anti-inflammatory, antimicrobial, analgesic, and antitumor effects for review. Several studies have shown antitumor effects of snake venoms or their isolated components [3–9]. CTX has been shown to inhibit proliferation of various cell lines (*in vitro*) and growth of various tumors* in vivo* [10]. As reported by Cura and colleagues [11], CTX is toxic to several tumor cell lines* in vitro* [7] and, in some of them, via epidermal growth factor receptors [12]. The antitumoral effects of CTX have also been reported in patients with lung and mammary carcinoma [8]. Evidence has been accumulated that CTX presents inhibitory effects on inflammatory response [13, 14]. Inflammation is closely associated with cancer growth [15]. In spite of the studies mentioned, the mechanisms involved in the antitumor effects of the CTX still remain to be determined.

Increased formation of prostaglandins PGE_2_ and PGD_2_ occurs in the beginning of an inflammatory response. Afterwards, the profile of lipid mediators activates the expression of 15-LOX in leukocytes, which switches the mediator profile of these cells from LTB_4_ to lipoxins (LXs). Lipoxin A_4_ (LXA_4_) and lipoxin B_4_ (LXB_4_) are synthesized by transcellular metabolism of AA due to an interaction among neutrophils, endothelial cells, fibroblasts, and platelets localized in the inflammatory exudate. Afterwards, the profile of lipid mediators switches from proinflammatory eicosanoids to lipoxins (LXs) that bind to G-protein-coupled LXA_4_ receptor (formyl peptide receptor 2-FPR2/ALX) and triggers the proinflammatory termination signal [16]. LXs are produced from arachidonic acid* via* 5-lipoxygenase (5-LO) and 15-lipoxygenase (15-LO) pathways [17]. Acetylation of cyclooxygenase-2 (COX-2) by aspirin leads to biosynthesis of 15-epi-lipoxins [18], the 15-epimers carbon of native LXs. 15-Epi-LXA_4_ has more potent and longer lasting effects than does the native LXA_4_ that is less rapidly inactivated [19, 20], for review. The native LXs and their stable analogues regulate cell functions through activation of G-protein-coupled LXA_4_ receptor (formyl peptide receptor, FPR2, also termed ALXR). These receptors are expressed by neutrophils and monocytes [21–24]. As FPRs are potentially important therapeutic targets, studies have been focused on identification of natural and synthetic compounds having the ability to interact with these receptors or interfere with the FRP-involved pathways [25, 26].

LXs are involved in the development of pathological conditions such as rheumatoid arthritis, asthma, sepsis, diabetes, and tumor [16, 19, 27]. Administration of LXs and their natural analogue 15-epi-LXA_4_ causes inhibition of disease-related inflammation and suppresses tumor growth and cancer-associated pain [20, 28–31]. CTX treatment promotes release of LXA_4_ and 15-epi-LXA_4_ in cultured macrophages and macrophages cocultivated with tumor cells, which may contribute to the antiproliferative activity of these leukocytes [32].

We tested herein the hypothesis that CTX treatment reduces tumor growth through formyl peptide receptors (FPRs) and production of LXA_4_ and 15-epi-LXA_4_. To investigate this hypothesis, we used the Walker 256 tumor model developed in the rat paw [33]. Injection of Walker 256 carcinoma cells results in the development of inflammation, cell proliferation, and tumor tissue growth, angiogenesis [34] and hyperalgesia [33].

Over 70% of anticancer compounds are either natural products or natural product-derived compounds [35]. The discovery of new drugs for different types of cancer is a hot area of investigation since many tumors still remain unresponsive to any existing treatment [36]. Evidence is presented herein that Crotoxin may be a new therapeutic drug to be clinically investigated so as to treat cancer. It has been shown in clinical trials that the LXA_4_ analogues present efficacy and safety [37], expanding the pharmacological perspectives herein proposed.

## 2. Material and Methods

### 2.1. Animals

Male Wistar rats, weighing between 160 and 180 g, were used throughout the study. The rats were housed in an animal care facility and taken to the testing room 2 days before the experiment. Food and water were available* ad libitum*. All experiments and assays were carried out in accordance with the guidelines for the ethical use of conscious animals in pain research, published by the International Association for the Study of Pain [38]. The Institutional Animal Care Committee of the Butantan Institute approved the procedures used in this study (CEUAIB, protocol number 359/2006).

### 2.2. Crotoxin (CTX)

CTX was obtained from lyophilised venom of* Crotalus durissus terrificus* supplied by the Laboratory of Herpetology, Butantan Institute, São Paulo, Brazil, and maintained at −20°C. Crude venom solution was subjected to anion-exchange chromatography as previously described by [39], using a Mono-Q HR 5/5 column in an FPLC system (Pharmacia, Uppsala, Sweden). The fractions (1 mL/min) were eluted using a linear gradient of NaCl (0-1 mol/L in 50 mmol/L Tris-HCl, pH 7.0). Three peaks (p1, p2, and p3) were obtained: p2 corresponded to the pure CTX fraction (about 60% of the crude venom); peaks 1 and 3 included the other CdtV toxins. Prior to pooling, the fractions containing CTX were tested for homogeneity by nonreducing sodium dodecyl sulphate-polyacrylamide gel electrophoresis (12.5%) [40] and the phospholipase A_2_ activity was assessed by a colorimetric assay using a synthetic chromogenic substrate [41].

### 2.3. Pharmacological Treatments

CTX was subcutaneously injected (18 *μ*g per rat in 300 *μ*L of the saline), daily, for 5 days. The dose of CTX was based on previous work [42] and did not cause clinical signs of* Crotalus durissus terrificus* envenomation, such as neurotoxic faces, external and internal ophthalmoplegia, and respiratory paralysis [43]. Other rats received LXA_4_ (2.0 *µ*g per rat/300 *µ*L saline, subcutaneously), based on Von Der Weid et al. [44]. The same volume of saline was subcutaneously administered to the respective reference groups. To investigate the involvement of FPRs in the CTX effect, rats were treated with Boc-2, a selective FPRs antagonist, butoxycarbonyl-Phe-Leu-Phe-Leu-Phe, from Phoenix Pharmaceutical Inc., USA, in a dose of 5 *μ*g per rat, intraperitoneally, in 1 mL saline containing 1% dimethyl sulfoxide [44]. Thirty minutes later, the animals were subcutaneously injected with CTX or LXA_4_ or saline, in the same volume. The results were compared to two reference groups; the first group received saline by the same route used for tumor cell inoculation and the other received LXA_4_. On the fifth day of the injection of tumor cells, the animals were submitted to analysis of tumor growth through increase in tumor volume, mechanical hyperalgesia and allodynia, in addition to plasma collection for measurement of the LXA_4_ and 15-epi-LXA_4_.

### 2.4. Walker 256 Carcinoma Cell Inoculation

After 5 days of intraperitoneal injection of Walker 256 carcinoma cells (1 × 10^7^/2 mL), ascitic liquid from the peritoneal cavity was collected to obtain fresh tumor cells and the percentage of viable cells was determined by using 1% Trypan blue aqueous solution in a Neubauer chamber. Cells were harvested and a suspension of 10^7^ cells per mL was obtained by dilution with phosphate-buffered saline (PBS, pH 7.4). Antibiotic (Benzylpenicillin, 120,000 units in 10 mL of cell suspension, Benzetacil; Eurofarma®, Brazil) was added to cell suspension to avoid microbial contamination. Tumor cells (100 *μ*L) were then subcutaneously injected into the plantar region of the rat right hind paw; PBS (100 *μ*L) injection into the contralateral hind paw was used as reference for tumor growth assessment. This was performed as described in our previous study [33].

### 2.5. Tumor Growth Assessment

Tumor growth was assessed with the aid of a pachymeter (Mitutoyo, Japan) or by measurement of the volume increase (edema) of paws up to the tibiotarsal articulation. The measurements were carried out before the injection of tumor cells and PBS (in the contralateral paw) and at chosen time intervals thereafter according to Brigatte et al. [33]. The percentage of volume increase was measured in each paw. The difference between values obtained for both paws was used as a measure of edema volume increase and so tumor growth.

### 2.6. Measurement of Plasma LXA_4_ and 15-Epi-LXA_4_ Levels

On the fifth day of the experiment, after the last measurement of the paw volume, the animals were anesthetized with ketamine (100 mg kg^−1^, 0.5 mL/kg, Vetbrands Brasil Ltda., Brazil) and xylazine (10 mg kg^−1^, 0.5 mL/kg, Vetbrands Brasil Ltda., Brazil), intraperitoneally injected, and blood samples were obtained from the abdominal aorta in tubes containing disodium salt of ethylene diamine tetra acetic acid (EDTA) as anticoagulant. LXA_4_ and 15-epi-LXA_4_ were measured in plasma by immunoenzymatic assays [32, 45] using specific kits for each LX (Neogen, Lexington, KY, USA). Plasma samples were acidified with 1 N HCl to pH 3.4–3.6 and passed slowly through an octadecylsilyl silica column (C18 Sep-Pak® column, Waters® Corporation, USA), prewashed with 10 mL absolute ethanol and 10 mL water. After activation of the column with 10 mL water, 2 mL absolute ethanol, and 2 mL water again, the eicosanoids were eluted from the column with 1 mL water, 1 mL ether, and 2 mL methyl formate and the samples dried under a stream of nitrogen. The sensitivity of the assay was of 20 pg/mL.

### 2.7. Histopathological Analysis

The animals were euthanized in a CO_2_ chamber, on the fifth day after tumor cell injection. The right hind paw was removed and fixed in 10% formalin. Samples were embedded in paraffin, sectioned into 5 *μ*m sections, and stained with monastral blue [46]. The number and diameter of vessels were then determined. Micrographs were taken in an Olympus BX 51 microscope (USA) and measurements were carried out using the Axio Vision 4.8 program.

### 2.8. Statistical Analysis

Statistical analysis of the differences between groups was performed according to Glantz [47] by using the GraphPad InStat software version 3.01 (GraphPad Software Inc., San Diego, CA, USA). One-way ANOVA followed by Bonferroni's test was also used to prepare dose-response curves for a single time point. *p* < 0.05 was considered for differences to be significant. The alpha level (significance level related to the probability of rejecting a true hypothesis) was set to 0.05. Significant differences were then compared using Bonferroni's test with a significance coefficient of 0.05. The results are presented as mean values ± standard error of means.

## 3. Results

### 3.1. CTX Inhibited the Edema Induced by Inoculation of Walker 256 Carcinoma Cells in the Plantar Region of the Rat Right Hind Paw, Decreased Formation of New Blood Vessels, and the Blood Vessel Diameters

**Figure 1:**
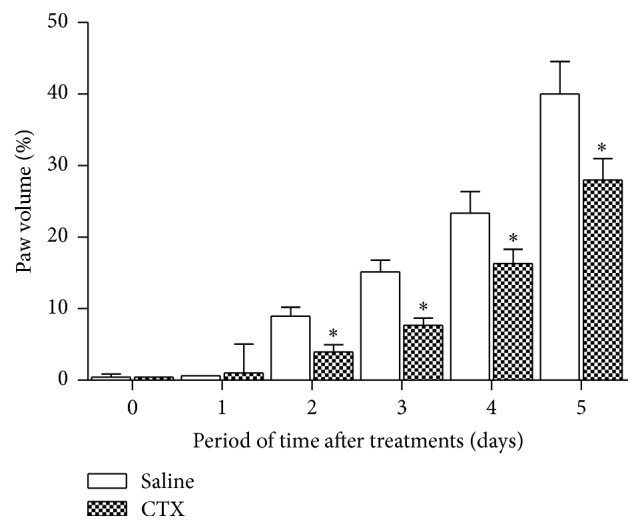
Effect of CTX on edema induced by Walker 256 carcinoma cell inoculation. Tumor cells (1 × 10^6^ in 100 *µ*L) were subcutaneously injected into the plantar region of the right hind paw of the rats. CTX (18 *µ*g per rat in 300 *µ*L) or saline (vehicle control) was s.c. administered daily, during 5 days (first injection on day 1, immediately after tumor cell inoculation). The increase in paw volume was determined in rat hind paws before and at different times after cell inoculation. Edema is expressed as percentage of volume increase in relation to the initial volume of the paw. Each point represents the mean ± SEM of 5 rats. ^*∗*^ *p* < 0.05, significantly different from mean values for saline injected animals at fifth day after cell injection.

**Figure 2:**
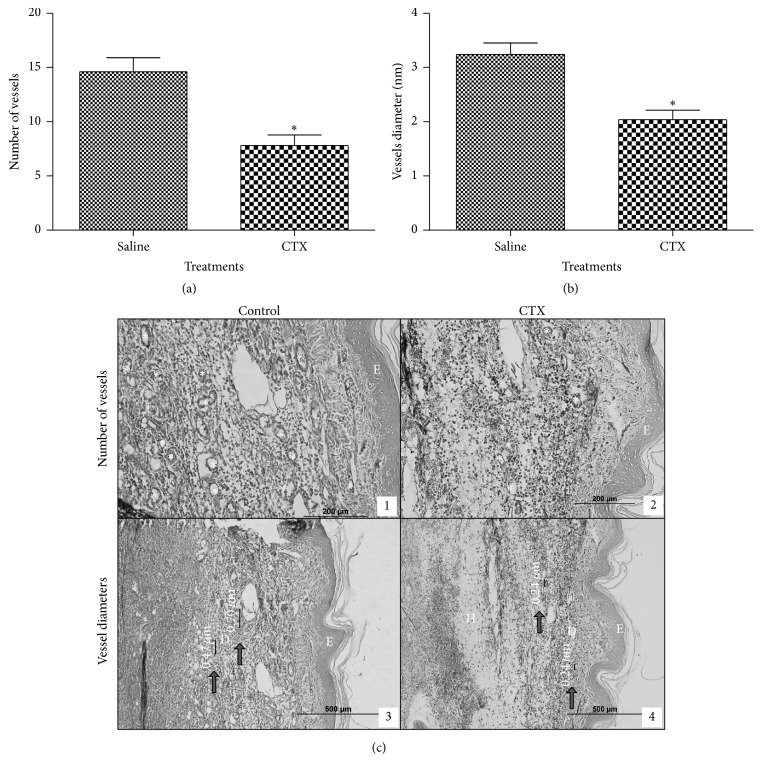
Effect of CTX on changes in number of vessels and vessel diameters induced by Walker 256 carcinoma cell inoculation. Tumor cells (1 × 10^6^ in 100 *µ*L) were subcutaneously injected into the plantar region of the right hind paw of the rats. CTX (18 *µ*g per rat in 300 *µ*L) or saline (vehicle control) was s.c. administered daily, during 5 days (first injection on day 1, immediately after tumor cell inoculation). (a) The number of vessels and (b) vessel diameters (nm) were determined on the fifth day. (c) Representative histopathological slides of paws injected with Walker 256 carcinoma cells of animals treated with saline (1 and 3) or CTX (2 and 4). Samples were obtained on the fifth day of treatment and stained with monastral blue. (*∗*) The number of vessels. (→) The vessel diameter. (E), (D), and (H) represent epidermis, dermis, and hypodermis, respectively. Detail of vessel diameters is indicated in (3) and (4) (400x). Each point represents the mean ± SEM of 5 rats. ^*∗*^ *p* < 0.05, significantly different from mean values for saline injected rats at the fifth day after cell injection.

The injection of tumor cells caused a significant and progressive increase in paw volume as compared to the values obtained before cell inoculation (Figure 1). The edema was measurable already on the second day (9%) after cell injection and reached up to 40% increase on the fifth day (Figure 1). Daily subcutaneous administration of CTX, for 5 days, caused significant decrease of paw volume from the third day of tumor cell inoculation (third day: 49%; fourth day: 30%; fifth day: 30%) (Figure 1). A representative histological slide of a normal rat paw is shown in Brigatte and colleague [33]. After 5 days of treatment with CTX, the number of vessels was significantly lowered (47%) as compared to PBS treated animals (Figures 2(a), 2(c)(1), and 2(c)(2)). Also, vessel diameters (Figures 2(b) and 2(c)(3) and 2(c)(4)) were significantly smaller (37%) in CTX treated rats.

### 3.2. CTX Led to an Increase of LXA_4_ and 15-Epi-LXA_4_ Plasma Levels

**Figure 3:**
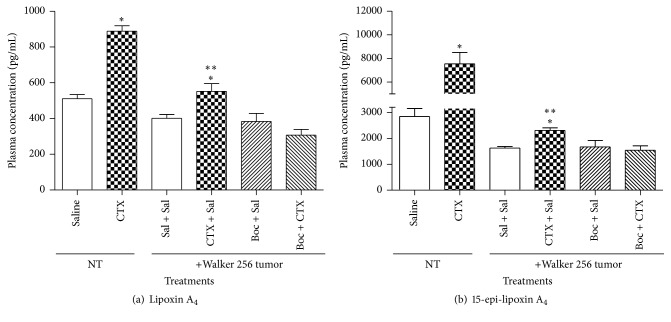
Effect of CTX on plasma levels of LXA_4_ and 15-epi-LXA_4_ in Walker 256 tumor-bearing rats. Not tumor-bearing (NT) or Walker 256 tumor-bearing rats were s.c. daily treated with saline (vehicle control) or CTX (18 *µ*g per rat), during 5 days (first injection on day 1, immediately after tumor cell inoculation or same volume of saline). Rats were also treated with Boc-2 (5 *μ*g per rat /1 mL, i.p.) or same volume of saline, daily, during 5 days, 30 minutes before the s.c. injection of CTX or saline. On the fifth day of treatments, the animals were anesthetized for the collection of blood and plasma was obtained to determine (a) LXA_4_ or (b) 15-epi-LXA_4_ levels. Each point represents the mean ± SEM of 5 rats. ^*∗*^ *p* < 0.05, significantly different from mean values for saline injected rats after cell injection. ^*∗∗*^ *p* < 0.05, significantly different from mean values for the Boc-2 + CTX group.

Plasma LXA_4_ concentration was assessed on the fifth day of CTX treatment (Figure 3(a)). Treatment of control (NT) rats with CTX induced a significant increase of LXA_4_ plasma levels (74%) when compared to saline treated animals. Walker 256 tumor-bearing animals showed low plasma concentrations of both LXA_4_ and 15-epi-LXA_4_. The treatment of tumor-bearing rats with the toxin, under the same experimental conditions described above, induced an increase of plasma levels of LXA_4_ and 15-epi-LXA_4_ (38%) when compared to saline (Figure 3(a)). CTX induced a significant increase in plasma concentration of the stable analogue 15-epi-LXA_4_ (by 1.65-fold) as compared to animals without tumor (NT) and injected with saline (Figure 3(b)). Similar increase was observed in Walker 256 tumor-bearing rats that received subcutaneous injection of the toxin (42%), daily, for five days as compared to saline injected tumor-bearing animals (Figure 3(b)).

### 3.3. Evidence That Formyl Peptide Receptors (FPRs) Are Involved in the Reducing Effects of CTX on Tumor Growth and Plasma Levels of LXA_4_ and 15-Epi-LXA_4_

The treatment of rats with no tumor (NT) with Boc-2 thirty minutes before the subcutaneous injection of CTX blocked the increase in plasma concentration of both LXA_4_ and 15-epi-LXA_4_ when compared to treatment with saline. These results show that the FPRs mediated the effects of CTX on production of both lipid mediators. The Boc-2* per se* did not cause marked changes in plasma levels of LXA_4_ and 15-epi-LXA_4_ when compared to saline injected rats (Figures 3(a) and 3(b)).

To evaluate the participation of LXA_4_ and 15-epi-LXA_4_ in the antitumoral effects of CTX, Boc-2 was i.p. administered, daily, 30 minutes before the subcutaneous injection of CTX for 5 days, from the 1st day of Walker 256 tumor cell injection.

**Figure 4:**
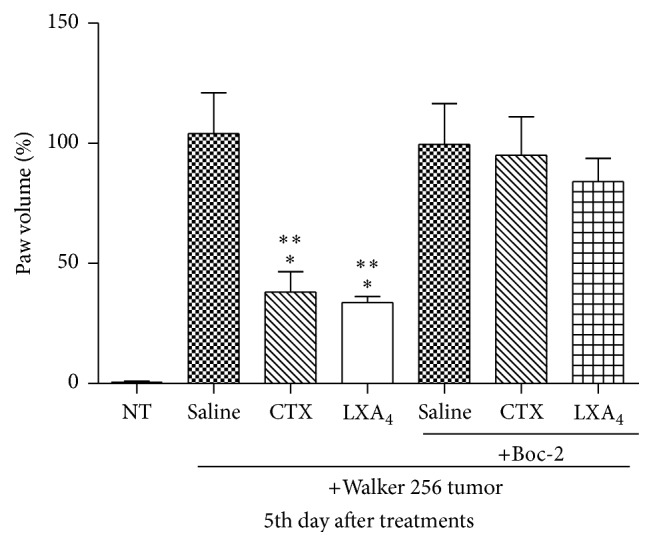
Effects of CTX and LXA_4_ on tumor growth. Not tumor-bearing (NT) or Walker 256 tumor-bearing rats were s.c. daily treated with saline (vehicle control) or CTX (18 *µ*g per rat) or LXA_4_ (2.5 *µ*g per rat/1 mL), during 5 days (first injection on day 1, immediately after tumor cell inoculation or same volume of saline). Rats were also treated with Boc-2 (5 *μ*g per rat/1 mL, i.p.) or the same volume of saline, daily, during 5 days, 30 minutes before the s.c. injection of CTX or saline. On day 5 of treatments, tumor growth was performed with aid of a micrometer, by volume increase (edema) of paws up to the tibiotarsal articulation. Each point represents the mean ± SEM of 5 rats. ^*∗*^ *p* < 0.05, significantly different from mean values for normal NT animals. ^*∗∗*^ *p* < 0.05, significantly different from mean values for rats injected with saline or Boc-2.

The results showed that, on the fifth day of the tumor inoculation, both CTX and LXA_4_ (a FPRs agonist) inhibited Walker 256 tumor growth (by 63% and 67%, resp.) (Figure 4). Concomitantly, some animals received Boc-2 + saline or Boc-2 + LXA_4_. It is noticeable that, on the 5th day of treatment, Boc-2 completely abolished the reducing effect of the toxin on tumor growth as compared to saline (Figure 4). The same was observed for LXA_4_ administration.

**Figure 5:**
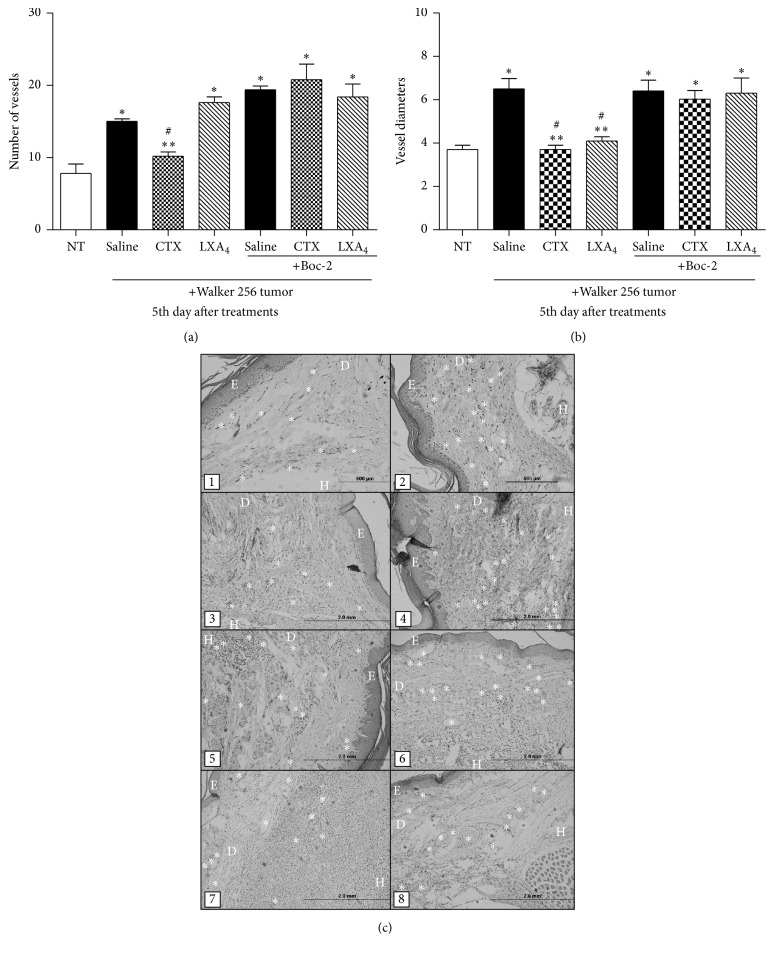
Effects of CTX and LXA_4_ on formation and diameters of blood vessels. NT animals or Walker 256 tumor-bearing rats were s.c. daily treated with saline (vehicle control) or CTX (18 *µ*g per rat) or LXA_4_ (2.5 *µ*g per rat/1 mL), during 5 days (first injection on day 1, immediately after tumor cell inoculation or same volume of saline). Rats were also treated with Boc-2 (5 *μ*g per rat/1 mL, i.p.) or the same volume of saline (control), daily, during 5 days, 30 minutes before the s.c. injection of CTX or saline. On the fifth day of treatments, the animals were euthanized to obtain the paws for histological analysis and determination of the (a) number of vessels and (b) vessel diameters. In (c), NT animal (1) and Walker 256 tumor-bearing rats treated with saline (2); CTX (3); LXA_4_ (4); Boc-2 + saline (5); Boc-2 + CTX (6); and Boc-2 + LXA_4_ (7, 8). (*∗*) Number of the vessels. The slides (E), (D), and (H) show epidermis, dermis, and hypodermis, respectively. Each point represents the mean ± SEM of 5 rats. ^*∗*^ *p* < 0.05 significantly different from mean values for NT rats. ^*∗∗*^ *p* < 0.05, significantly different from mean values for saline injected rats. ^#^ *p* < 0.05, significantly different from mean values for Boc-2 treated rats.

The pretreatment with Boc-2 also blocked the inhibitory effect of CTX on formation of new vessels (Figure 5(a)). The treatment with LXA_4_ did not interfere with the formation of vessels as compared to saline. On the other hand, the diameter of the vessels was decreased by both CTX and LXA_4_ and this effect was totally abolished by Boc-2 (Figures 5(a) and 5(b)). Tissue histological slides of the paws (Figure 5(c)) were obtained from NT animal (1) and Walker 256 tumor-bearing rats treated with saline (2); CTX (3); LXA_4_ (4); Boc-2 + saline (5); Boc-2 + CTX (6); and Boc-2 + LXA_4_ (7, 8).

**Figure 6:**
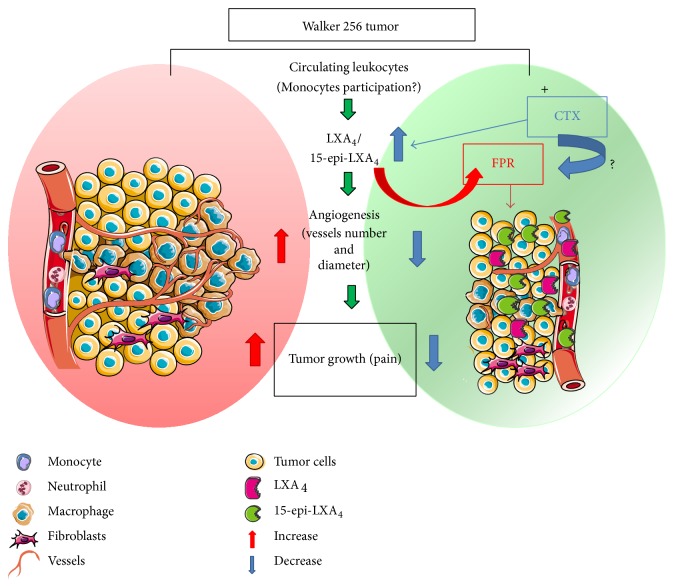
Proposed scheme for CTX action on Walker 256 tumor growth suppression. Subcutaneous injection of Walker 256 carcinoma cells in the plantar region of the rat right hind paw promoted a marked infiltration of leukocytes into the deep dermis that migrated from the systemic circulation. Five days after tumor cell injection, there was a marked proliferation of tumor cells. Monocyte chemotactic factors or extracellular matrix proteins are secreted by solid tumor, which attract and activate macrophages. Animals treated with CTX showed increased plasma levels of LXA_4_ and its analogue being probably released by leukocytes (mainly macrophages but also neutrophils and monocytes). The increased formation of LXA_4_ and 15-epi-LXA_4_ is accompanied by tumor growth reduction and a significant decrease in both number and diameter of vessels and therefore pain attenuation. CTX actions require the participation of FPRs.

Together, the data presented here suggest that the CTX induced increased plasma levels of LXA_4_ and its analogue being probably released by leukocytes into the deep dermis that migrated from the systemic circulation, which might inhibit both tumor growth and formation of new vessels via FPRs. This proposal is summarized schematically in Figure 6.

## 4. Discussion

Tumor growth induced by intraplantar inoculation of Walker 256 carcinoma cells in rats was assessed by the increase in volume of the glabrous region of the hind paw. The volume of the cell inoculated paw started to increase on the second day after inoculation, and thereafter it increased progressively up to the fifth day as observed in our previous work [33]. The features of cancer pain symptoms (including hyperalgesia and allodynia) and of tissue morphological changes observed herein were also reported in our previous study [33].

CTX significantly inhibited tumor growth from the second day and this inhibition remained until the fifth day of study. This effect was accompanied by a decrease in formation of new vessels and in the diameter of the vessels, suggesting that CTX interferes with tumor growth by impairing angiogenesis.

Recent results have shown that CTX inhibits proliferation of human leukemic Jurkat T-cell line (Sandra Coccuzzo Sampaio and Yara Cury, unpublished data) and LLC WRC 256 tumor cells (Odair Jorge Faiad and Sandra Coccuzzo Sampaio, unpublished data). CTX also inhibits t.End.1 cell function, indicating direct action of this toxin on endothelial cells [48]. These observations are not due to alterations in cell viability. In addition to this direct activity of the toxin on tumor and endothelial cells, CTX raises the production of reactive oxygen and nitrogen species and LXA_4_ and its analogue 15-epi-LXA_4_ in macrophages cocultured with LLC WRC 256 tumor cells, reducing tumor cell proliferation. This inhibitory action is suppressed by blocking the formyl peptide receptors using Boc-2 [32].

CTX significantly inhibited tumor growth from the second day and this inhibition remained until the fifth day of study. This effect was accompanied by a decrease in formation of new vessels and in the diameter of the vessels, suggesting that CTX inhibition of tumor growth involves impairment in angiogenesis. Angiogenesis has been associated with the development of several diseases such as rheumatoid arthritis, psoriasis, and cancer [49]. Tumor growth depends on a persistent neovascularization [50, 51] and is proportional to the extent of angiogenesis. Inhibition of angiogenesis causes tumor regression [52, 53]. Tumor angiogenesis is a combination of angiogenesis and vasculogenesis and the mainstay of tumor blood vessels derived from preexisting ones, although the circulating endothelial precursor cells contribute to the growth of endothelial cell mass [54]. Active proliferation of tumor cells, which usually accompanies the initial phase of tumor growth, is balanced by the cell death caused by the withdrawal of blood supply to the tumor. Rapid and exponential tumor growth requires neovascularization whereas angiogenesis is paralleled to the process of metastasis [55, 56]. We demonstrated in previous studies that CTX inhibits secretory activity and endothelial cell function, evidencing direct action of this toxin on endothelial cells [48]. In addition, macrophages treated with CTX inhibit* in vitro* angiogenic events and the consequent formation of capillary structures by endothelial cells in 3D matrix [57].

An inflammation state is established in association with solid tumor growth [15]. Monocytes are recruited from the systemic circulation into tumor tissue, in response to chemokines secreted by tumor cells, and differentiate into macrophages [58, 59]. Tumor-associated macrophages modulate tumor cell migration, extravasation, and also angiogenesis [60, 61]. Activated macrophages exert tumoricidal effect by secreting molecules such as hydrogen peroxide (H_2_O_2_), nitric oxide (NO), and LXs [20, 62–64].

Various leukocytes including monocytes, polymorphonuclear cells, and macrophages secrete LXA_4_ and 15-epi-LXA_4_ [20, 32, 62]. LXs are biosynthesised and rapidly inactivated, whereas related compounds, such as 15-epi-LXA_4_, are more stable [65]. Acetylation of cyclooxygenase-2 induced by aspirin or other endogenous substrates (cytochrome p450 and reactive oxygen species) leads to stereoselective formation (40%* R* and 60%* S* form) of 15-epi-lipoxins that are more potent and longer acting than the native 15-S containing LX form [16, 18, 66]. Treatment with CTX caused a significant increase in plasma concentrations of LXA_4_ and its stable analogue in both control and tumor-bearing rats. Similar results were previously published [67, 68]. Despite the direct actions of CTX on cells of the tumor microenvironment, such as tumor and endothelial cells, production of LXA_4_ and its analogue by macrophages might also be involved in the inhibitory action of the toxin on tumor growth and angiogenesis herein reported.

The actions of these lipid mediators were mediated by FPRs since pretreatment with Boc-2, antagonist of the FPR2/ALX, and FPR1 [67, 68] completely blocked it. Similar observations have been previously published [67, 68].

Only CTX decreased the number of vessels whereas both CTX and LXA_4_ decreased vessel diameters. Chen and colleagues [29] reported an inhibitory effect of LXA_4_ and 15-epi-LXA_4_ on primary tumor growth. However, the analogue was able to induce toxicity of tumor cells whereas LXA_4_ did not cause antiproliferative effects [29]. CTX induced formation of the analogue in large quantities, which probably led to inhibition of angiogenesis and so tumor growth. The inhibitory action of CTX and LXA_4_ on paw volume increase, observed at the fifth day after injection of tumor cells, was completely abolished by Boc-2. Lipoxins and their stable analogues exert biological actions, such as anti-inflammatory and antiangiogenic properties, by binding to FPRs and then interfering with cell proliferation and tumor growth [31, 37, 69–71].

The reduction of tumor mass induced by treatment with the toxin was associated with a decrease of the hyperalgesic response (40%) (*data not shown*). This antinociceptive effect of CTX was not mediated by opioids, since naloxone, a nonspecific opioid receptor antagonist, did not modify it. Evidence has been accumulated that LXs and their analogues promote analgesic effects in bone cancer through reduction in proinflammatory mediators [30]. Nogueira-Neto and colleagues [72] showed that CTX induces a long-lasting antinociceptive effect in neuropathic pain, induced by transection of rat sciatic nerve, via central muscarinic, *α*-adrenergic, and serotonergic receptors. 5-Lipoxygenase-derived lipid mediators are involved in the modulation of this effect. Therefore, the analgesic effect observed on day 5 may be a result of both reduction of the tumor mass* per se* and the analgesic activity described for LXs. This proposition is reinforced by data demonstrating the ability of LXs to modulate the events involved in tumor growth and cancer pain [29–31].

In conclusion, CTX, the main neurotoxic component of* Crotalus durissus terrificus* venom, reduced Walker 256 tumor growth possibly due to an antiangiogenic effect. LXA_4_ and 15-epi-LXA_4_ are involved in the antitumor effects of CTX. The FPRs played a key role in the effect of the CTX on tumor growth. These receptors mediated the increase in plasma levels of LXA_4_ and 15-epi-LXA_4_ and also the actions of the lipid mediators.

## Additional Points

Highlights of this paper are as follows:

- Crotoxin, South American rattlesnake (*Crotalus durissus terrificus*) venom.
- Antitumoral activity.
- Mechanisms of action: plasma concentrations of lipoxin A_4_ and its natural analogue 15-epi-LXA_4_, LXA_4_/15-epi-LXA_4_ release; inhibition of both tumor growth and formation of new vessels via FPRs.
